# Prognostic factors for invasive mucinous adenocarcinoma of the lung: systematic review and meta-analysis

**DOI:** 10.1186/s12957-024-03326-4

**Published:** 2024-02-02

**Authors:** Ting Zhao, Jianhua Yi, Dan Luo, Junjun Liu, Xianming Fan, Qibiao Wu, Wenjun Wang

**Affiliations:** 1 Department of Respiratory and Critical Care Medicine, The Affiliated Hospital, Southwest Medical University, 646099 Luzhou, Sichuan China; 2 Faculty of Chinese Medicine, State Key Laboratory of Quality Research in Chinese Medicine and University Hospital, Macau University of Science and Technology, Taipa, 999078 Macao China; 3 Zhuhai MUST Science and Technology Research Institute, 51900 Zhuhai, Guangdong China; 4 Inflammation & Allergic Diseases Research Unit, The Affiliated Hospital, Southwest Medical University, 646099 Luzhou, Sichuan China

**Keywords:** Invasive mucinous adenocarcinoma of the lung, Prognostic factors, Meta-analysis, Systematic review

## Abstract

**Background:**

Invasive mucinous adenocarcinoma of the lung (IMA) is a unique and rare subtype of lung adenocarcinoma with poorly defined prognostic factors and highly controversial studies. Hence, this study aimed to comprehensively identify and summarize the prognostic factors associated with IMA.

**Methods:**

A comprehensive search of relevant literature was conducted in the PubMed, Embase, Cochrane, and Web of Science databases from their inception until June 2023. The pooled hazard ratio (HR) and corresponding 95% confidence intervals (CI) of overall survival (OS) and/or disease-free survival (DFS) were obtained to evaluate potential prognostic factors.

**Results:**

A total of 1062 patients from 11 studies were included. In univariate analysis, we found that gender, age, TNM stage, smoking history, lymph node metastasis, pleural metastasis, spread through air spaces (STAS), tumor size, pathological grade, computed tomography (CT) findings of consolidative-type morphology, pneumonia type, and well-defined heterogeneous ground-glass opacity (GGO) were risk factors for IMA, and spiculated margin sign was a protective factor. In multivariate analysis, smoking history, lymph node metastasis, pathological grade, STAS, tumor size, and pneumonia type sign were found to be risk factors. There was not enough evidence that epidermal growth factor receptor (EGFR) mutations, anaplastic lymphoma kinase (ALK) mutations, CT signs of lobulated margin, and air bronchogram were related to the prognosis for IMA.

**Conclusion:**

In this study, we comprehensively analyzed prognostic factors for invasive mucinous adenocarcinoma of the lung in univariate and multivariate analyses of OS and/or DFS. Finally, 12 risk factors and 1 protective factor were identified. These findings may help guide the clinical management of patients with invasive mucinous adenocarcinoma of the lung.

**Supplementary Information:**

The online version contains supplementary material available at 10.1186/s12957-024-03326-4.

## Introduction

Lung cancer is still the leading cause of cancer death worldwide. Lung cancer can be divided into two major categories: non-small cell lung cancer and small cell lung cancer. Lung adenocarcinoma is the most common type of non-small cell lung cancer [1]. According to the new classification by the European Respiratory Society (ERS), American Thoracic Society (ATS) and International Association for Cancer Research (IASLC) in 2011, and the World Health Organization standard in 2015, invasive mucinous adenocarcinoma of the lung is a unique and rare subtype of adenocarcinoma of the lung, accounting for approximately 2–10% [2–4]. The pathological characteristic is that the tumor cells are composed of goblet or columnar cells, and the nucleus is located at the base, with a wide range of cytoplasmic mucins and a variety of growth patterns, including squama wings, acini, mastoid head, and micropapillary [5, 6].

According to the current study, the prognosis of IMA is controversial compared with that of nonmucinous lung adenocarcinoma. Some studies have shown that IMA has a poor survival outcome, while other studies have shown different results [7, 8]. Compared with other subtypes of lung adenocarcinoma, IMA has significantly different clinicopathological features, molecular phenotypes, and radiological features [9, 10]. At present, many factors may be potential prognostic factors for IMA. However, as IMA accounts for only 2–10% of lung adenocarcinomas, the number of patients in most studies is relatively small. Meanwhile, there is no comprehensive systematic evaluation of the prognostic factors for IMA. There is still some controversy about the prognostic relevance of these factors.

The inconsistent results among different studies may be caused by many factors, such as sample size, study design, and basic patient characteristics. A single study cannot confirm the prognosis of certain factors for IMA. Therefore, it is necessary to carry out a systematic review and meta-analysis. This study aims to comprehensively assess all factors that possibly affect the prognosis of IMA and provide more comprehensive evidence for the clinical management of patients.

## Materials and methods

### Study guideline and search strategy

This meta-analysis was conducted according to the Preferred Reporting Items for Systematic Reviews and Meta-Analyses (PRISMA) guidelines [11]. The PRISMA checklist is shown in Additional file 1. The protocol registration number of this meta-analysis is INPLASY202410015. The link of the protocol is https://inplasy.com/inplasy-2024-1-0015/.

The PubMed, Embase, Cochrane, and Web of Science databases were searched for all English studies published from their inception to June 2023 by two authors. The following search terms were used: “invasive mucinous adenocarcinoma,” “lung,” “prognosis,” and “prognostic.” The detailed search strategy of each database is shown in Additional file 2.

### Selection criteria

All articles were selected based on the following inclusion criteria: (1) Patients with pathologically or histologically confirmed IMA; (2) cohort studies or case–control studies published between the establishment of the database and June 2023; (3) studies assessing the correlation of gender, age at the time of diagnosis, TNM stage, smoking status, metastasis status, genetic inheritance status, pathological characteristics and CT manifestations, and prognosis through OS and/or DFS (data with at least one prognostic factor); and (4) studies that provided sufficient information for extraction or estimation of the HR and 95% CI of OS and/or DFS. Studies were excluded if any of the following factors were identified: (1) Inadequate data for calculating the HR and 95% CI, (2) invasive mucinous adenocarcinoma of the lung secondary to other neoplasms, or (3) reviews, case reports, abstracts, animal studies, and unpublished or ongoing trials.

### Data extraction and quality assessment

Two investigators independently reviewed all eligible articles and extracted and recorded the required data using standardized forms. The following data were extracted: first author, year of publication, country of the study population, sample size, type of study, follow-up time, date of enrollment, HR, and 95% CI in univariate and multivariate analyses of OS and/or DFS.

The methodological quality of the included studies was assessed independently by two reviewers using the Newcastle–Ottawa Quality Assessment Scale (NOS). A study could score up to a maximum of nine stars based on the selection of subjects, comparability between groups, and measurement of exposure factors, which was 8–9 points for high quality, 5–7 points for medium quality, and less than 5 points for low quality [12].

### Statistical analysis

All statistical analyses were performed with STATA software version 15.1. The HRs and 95% CIs were used to assess the association of these prognostic markers (gender, age, TNM stage, smoking status, metastasis, genetic status, pathological features, and CT signs) with OS and/or DFS. *P* < 0.05 was considered statistically significant. We used *I*^2^ to assess heterogeneity among studies [13]. When heterogeneity existed (*P* < 0.05 or *I*^2^ > 50%), a random effects model was used; otherwise, a fixed effects model was used. However, heterogeneity might be influenced by multiple factors, and there was no indication that this value was sufficient for further analysis. Reportedly, low and intermediate values (*I*^2^ < 75%) could be acceptable because *I*^2^ results in small meta-analyses tend to be inaccurate [14, 15]. Only when ≥ 10 studies were included could further heterogeneity analysis be conducted [14].

When a meta-analysis contained at least 10 clinical trials, the potential publication bias was calculated by using funnel plots and Egger’s test [16–18].

## Results

### Search results

**Fig. 1:**
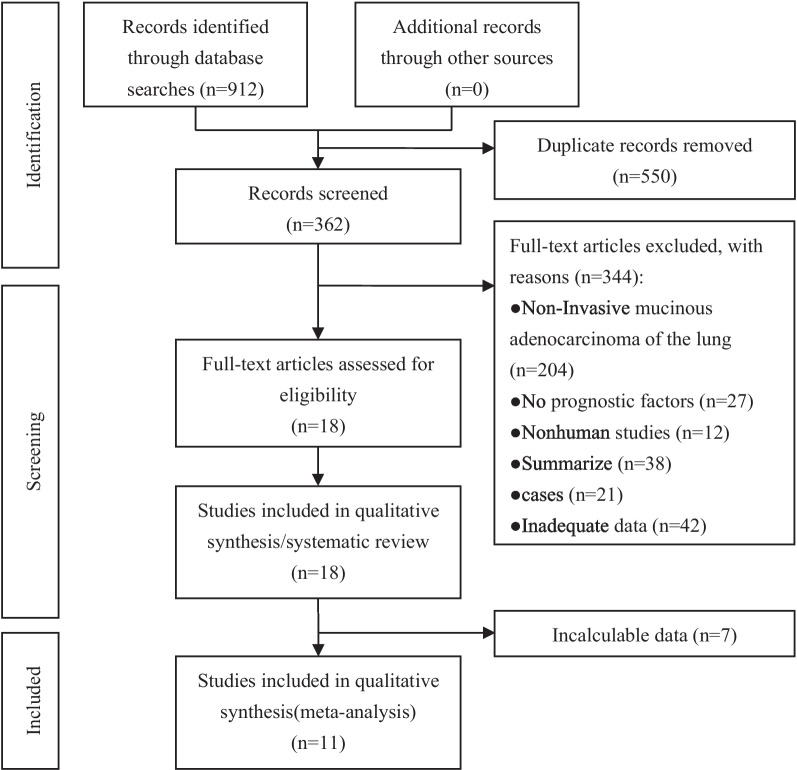
Flow diagram summarizing the reference search and study selection

A total of 912 eligible studies were retrieved from the initial search of the database. After 550 duplicate articles were excluded, 362 studies remained, 344 unrelated articles were removed after reading the titles and abstracts, and the last 7 articles were further excluded because of the lack of sufficient data after reading the full text. Finally, 11 articles met the selection criteria and were included in this meta-analysis (Fig. 1).

### Study characteristics

Table 1 summarizes the characteristics of the included studies. The 11 studies from Asian populations were retrospective studies published between 2016 and 2022, with sample sizes ranging from 26 to 317. A total of 1062 patients were included in the study. The NOS scores ranged from 6 to 8, indicating a moderate to high quality of the included studies.

**Table 1:** Main characteristics of the included studies

Study	Region	Study design	Sample	Follow-up (month)	Recruitment period	Survival endpoints	NOS	Data sources (R/C)
Gengpeng Lin (2018) [19]	China	Retrospective	26	NR	1999–2011	OS	7	R
Jian Wang (2022) [20]	China	Retrospective	98	Median 42	2013–2019	OS, DFS	8	R
Xiaoling Xu (2021) [21]	China	Retrospective	31	NR	2010–2015	OS	6	R
Takuya Matsui (2021) [22]	Japan	Retrospective	72	Median 63.3	2005–2015	OS, DFS	7	R
Daisuke Ueda (2021) [23]	Japan	Retrospective	47	Median 55	1997–2018	OS	8	R
Lei Cai (2021) [24]	China	Retrospective	51	NR	2010–2019	DFS	6	R
Tingting Wang (2021) [25]	China	Retrospective	317	Median 52.8	2011–2015	OS, DFS	8	R
Ho Yun Lee (2016) [26]	Korea	Retrospective	81	Median 33.7	2003–2011	OS, DFS	8	R
Tomonari Oki (2019) [27]	Japan	Retrospective	99	Median 55	2004–2017	OS	7	R
Hyun Jung Yoon (2022) [28]	Korea	Retrospective	121	Median 81.4	1998–2012	OS, DFS	7	R
Min A. Lee (2020) [29]	Korea	Retrospective	119	Median 89.3	1998–2012	OS	7	R

*Abbreviations*: *OS* overall survival, *DFS* disease-free survival, *NR* not reported, *R* reported, *C* calculated, *NOS* Newcastle–Ottawa Quality Assessment Scale

### Prognostic factors

Tables 2 and 3 show the pooled results for 17 potential prognostic factors in univariate and multivariate analyses, including the number of included studies, sample size, pooled HR, 95% CI, pooled *P*-value, and *I*^2^ value. Forest plots are shown in Additional file 3.

**Table 2:** The pooled results of the univariate analysis of OS and DFS

Clinical endpoints	Prognostic factors	No. of study	Sample	HR (95% *CI*)	*p*-value	*I*^2^ (%)
OS	Gender (female vs. male)	7	711	0.53 (0.40, 0.71)	0.001	0.0
	Age (older vs. younger)	8	830	1.10 (0.99, 1.22)	0.087	74.7
	TNM stage (III-IV vs. I-II)	2	98	3.62 (1.85, 7.08)	< 0.001	30.9
	Smoking status (yes vs. no)	6	801	1.82 (1.34, 2.47)	< 0.001	0.1
	Tumor size (bigger vs. smaller)	4	586	1.46 (1.10, 1.93)	0.008	82.0
	Metastasis (no vs. yes)					
	Lymph node metastasis	6	780	0.26 (0.19, 0.36)	< 0.001	24.7
	Pleural invasion	2	415	0.39 (0.23, 0.66)	< 0.001	0.0
	Pathological features					
	STAS (+ vs. −)	3	339	2.23 (1.16, 4.26)	0.015	0.0
	Pathological grade (higher vs. lower)	2	191	2.77 (0.91, 8.41)	0.072	53.8
	Gene mutation (positive vs. negative)					
	EGFR mutation	3	152	0.70 (0.35, 1.41)	0.322	0.0
	ALK mutation	2	129	0.62 (0.13, 3.03)	0.553	60.0
	CT manifestations					
	CT morphology (consolidative vs. nodular type)	2	240	3.11 (1.90, 5.11)	< 0.001	0.0
	Pneumonia type (pneumonia vs. solitary)	3	436	5.81 (3.68, 9.19)	< 0.001	0.0
	Well-defined heterogeneous GGO (+ vs. −)	2	240	2.31 (1.27, 4.19)	0.006	0.0
	Spiculated margin (+ vs. −)	2	240	0.70 (0.54, 0.92)	0.012	0.0
	Lobulated margin (+ vs. −)	2	240	1.09 (0.66, 1.80)	0.722	0.0
	Air bronchogram (+ vs. −)	3	557	1.40 (0.71, 2.78)	0.336	64.8
DFS	Gender (female vs. male)	3	536	0.67 (0.49, 0.90)	0.009	26.2
	Age (older vs. younger)	3	536	1.04 (1.01, 1.07)	0.013	0.0
	TNM stage (III-IV vs. I-II)	/	/	/	/	/
	Smoking status (yes vs. no)	3	536	1.41 (1.03, 1.93)	0.032	0.0
	Tumor size (bigger vs. smaller)	3	487	2.50 (0.91, 6.87)	0.076	90.5
	Metastasis (no vs. yes)					
	Lymph node metastasis	3	536	0.26 (0.19, 0.35)	< 0.001	33.6
	Pleural invasion	2	415	0.55 (0.25, 1.22)	0.144	55.6
	Pathological features					
	STAS (+ vs. −)	/	/	/	/	/
	Pathological grade (higher vs. lower)	2	123	4.46 (1.92, 10.38)	0.001	0.0
	Gene mutation (positive vs. negative)					
	EGFR mutation	2	172	0.52 (0.09, 2.91)	0.459	72.5
	ALK mutation	/	/	/	/	/
	CT manifestations					
	CT morphology (consolidative vs. nodular type)	/	/	/	/	/
	Pneumonia type (pneumonia vs. solitary)	2	389	5.17 (3.31, 8.08)	< 0.001	37.4
	Well-defined heterogeneous GGO (+ vs. −)	/	/	/	/	/
	Spiculated margin (+ vs. −)	/	/	/	/	/
	Lobulated margin (+ vs. −)	/	/	/	/	/
	Air bronchogram (+ vs. −)	2	438	1.77 (0.55, 5.67)	0.340	85.1

**Table 3:** The pooled results of the multivariate analysis of OS and DFS

Clinical endpoints	Prognostic factors	No. of study	Sample	HR (95% *CI*)	*p*-value	*I*^2^ (%)
OS	Gender (female vs. male)	3	176	0.80 (0.32, 1.96)	0.618	0.0
	Age (older vs. younger)	6	497	1.06 (0.99, 1.14)	0.090	66.6
	TNM stage (III-IV vs. I-II)	/	/	/	/	/
	Smoking status (yes vs. no)	4	385	4.71 (1.52, 14.58)	0.007	56.8
	Tumor size (bigger vs. smaller)	5	667	1.27 (1.07, 1.50)	0.006	67.9
	Metastasis (no vs. yes)					
	Lymph node metastasis	3	338	0.14 (0.07, 0.28)	< 0.001	0.0
	Pleural invasion	/	/	/	/	/
	Pathological features					
	STAS (+ vs. −)	2	240	6.10 (2.17, 17.12)	0.001	0.0
	Pathological grade (higher vs. lower)	2	191	3.44 (0.80, 14.75)	0.096	62.1
	Gene mutation (positive vs. negative)					
	EGFR mutation	/	/	/	/	/
	ALK mutation	2	129	0.44 (0.04, 4.49)	0.492	76.4
	CT manifestations					
	CT morphology (consolidative vs. nodular type)	3	321	3.44 (0.78, 15.17)	0.102	62.9
	Pneumonia type (pneumonia vs. solitary)	2	119	3.39 (1.19, 9.62)	0.022	3.4
	Well defined heterogeneous GGO (+ vs. −)	2	240	1.20 (0.44, 3.25)	0.722	0.0
	Spiculated margin (+ vs. −)	/	/	/	/	/
	Lobulated margin (+ vs. −)	/	/	/	/	/
	Air bronchogram (+ vs. −)	/	/	/	/	/
DFS	Gender (female vs. male)	/	/	/	/	/
	Age (older vs. younger)	3	300	1.02 (0.99, 1.06)	0.232	37.3
	TNM stage (III-IV vs. I-II)	/	/	/	/	/
	Smoking status (yes vs. no)	2	179	1.83 (0.84, 4.01)	0.130	0.0
	Tumor size (bigger vs. smaller)	3	251	2.22 (1.01, 4.90)	0.047	72.1
	Metastasis (no vs. yes)					
	Lymph node metastasis	2	219	0.18 (0.09, 0.35)	< 0.001	0.0
	Pleural invasion	/	/	/	/	/
	Pathological features					
	STAS (+ vs. −)	/	/	/	/	/
	Pathological grade (higher vs. lower)	2	123	8.94 (3.10, 25.80)	< 0.001	0.0
	Gene mutation (positive vs. negative)					
	EGFR mutation	/	/	/	/	/
	ALK mutation	/	/	/	/	/
	CT manifestations					
	CT morphology (consolidative vs. nodular type)	2	202	1.32 (0.15, 11.74)	0.804	79.3
	Pneumonia type (pneumonia vs. solitary)	2	389	2.90 (1.55, 5.42)	0.001	19.3
	Well defined heterogeneous GGO (+ vs. −)	/	/	/	/	/
	Spiculated margin (+ vs. −)	/	/	/	/	/
	Lobulated margin (+ vs. −)	/	/	/	/	/
	Air bronchogram (+ vs. −)	/	/	/	/	/

#### Gender

Seven studies evaluated the association of gender [19–21, 23, 25, 27, 28]. In univariate analysis, the OS (*HR*: 0.53; 95% *CI*: 0.40–0.71; *P* = 0.001; *I*^2^ = 0%) and DFS (*HR*: 0.67; 95% *CI*: 0.49–0.90; *P* = 0.009; *I*^*2*^ = 26.2%) of women were significantly better than those of men. In the multivariate analysis, there was no significant difference in OS between the two groups (*HR*: 0.80; 95% *CI*: 0.32–1.96; *P* = 0.618; *I*^2^ = 0%).

#### Age

Nine studies were included in the OS and DFS analyses based on age [19–21, 23, 25–29]. In univariate analysis, older patients had worse DFS than younger patients (*HR*: 1.04; 95% *CI*: 1.01–1.07; *P* = 0.013; *I*^2^ = 0%). Nevertheless, the difference in OS between the two groups was not significant (*HR*: 1.10; 95% *CI*: 0.99–1.22; *P* = 0.087; *I*^2^ = 74.7%). In the multivariate analysis, the pooled results of OS (*HR*: 1.06; 95% *CI*: 0.99–1.14; *P* = 0.090; *I*^2^ = 66.6%) and DFS (*HR*: 1.02; 95% *CI*: 0.99–1.06; *P* = 0.232; *I*^*2*^ = 37.3%) showed no significant difference.

#### TNM stage

Two studies were included in the analysis [19, 22]. The aggregated results showed that the OS of patients with stages III–IV disease was lower than that of patients with stages I–II disease in univariate analysis (*HR*: 3.62; 95% *CI*: 1.85–7.08; *P* < 0.001; *I*^2^ = 30.9%).

#### Smoking status

Seven studies provided relevant data [20, 23, 25–29]. In univariate analysis, the OS (*HR*: 1.82; 95% *CI*: 1.34–2.47; *P* < 0.001; *I*^2^ = 0.1%) and DFS (*HR*: 1.41; 95% *CI*: 1.03–1.93; *P* = 0.032; *I*^2^ = 0%) of smoking patients were shorter than those of nonsmoking patients. In the multivariate analysis, the trend of OS (*HR*: 4.71; 95% *CI*: 1.52–14.58; *P* = 0.007; *I*^2^ = 56.8%) was similar to that in the univariate analysis. However, for DFS (*HR*: 1.83; 95% *CI*: 0.84–4.01; *P* = 0.13; *I*^2^ = 0%), the merged result was not statistically significant in the multivariate analysis.

#### Tumor size

Five studies evaluated the association between tumor size and IMA [20, 22, 25–27]. In univariate analysis, the patients with larger tumors (*HR*: 1.46; 95% *CI*: 1.10–1.93; *P* = 0.008; *I*^2^ = 82.0%) had a worse prognosis than those with smaller tumors for OS. However, there was no significant difference in DFS between the two groups (*HR*: 2.50; 95% *CI*: 0.91–6.87; *P* = 0.076; *I*^2^ = 90.5%). In the multivariate analysis, patients with larger tumors had shorter OS (*HR*: 1.27; 95% *CI*: 1.07–1.50; *P* = 0.006; *I*^2^ = 67.9%) and DFS (*HR*: 2.22; 95% *CI*: 1.01–4.90; *P* = 0.047; *I*^2^ = 72.1%).

#### Lymph node metastasis

Six studies on lymph node metastasis were included [19, 20, 25, 27–29]. In univariate (*HR*: 0.26; 95% *CI*: 0.19–0.36; *P* < 0.001; *I*^2^ = 24.7%) and multivariate (*HR*: 0.14; 95% *CI*: 0.07–0.28; *P* < 0.001; *I*^2^ = 0%) analyses, the OS of patients with lymph node invasion was shorter than that of patients without lymph node invasion. A similar trend was observed for DFS in univariate (*HR*: 0.26; 95% *CI*: 0.19–0.35; *P* < 0.001; *I*^2^ = 33.6%) and multivariate (*HR*: 0.18; 95% *CI*: 0.09–0.35; *P* < 0.001; *I*^2^ = 0%) analyses.

#### Pleural invasion

Two studies provided relevant data [20, 25]. In univariate analysis, patients with pleural invasion had a worse prognosis than those without pleural invasion based on OS (*HR*: 0.39; 95% *CI*: 0.23–0.66; *P* < 0.001; *I*^2^ = 0.0%). Nevertheless, the pooled results for the DFS had no statistical significance (*HR*: 0.55; 95% *CI*: 0.25–1.22; *P* = 0.144; *I*^2^ = 55.6%).

#### STAS

Three articles evaluated whether STAS could be used as a prognostic marker for IMA [27–29]. In univariate (*HR*: 2.23; 95% *CI*: 1.16–4.26; *P* = 0.015; *I*^2^ = 0.0%) and multivariate (*HR*: 6.10; 95% *CI*: 2.17–17.12; *P* = 0.001; *I*^2^ = 0.0%) analyses, the OS of STAS-positive patients was shorter.

#### Pathological grade

Three studies evaluated the correlation between pathological grade and IMA [22, 24, 29]. In univariate (*HR*: 4.46; 95% *CI*: 1.92–10.38; *P* = 0.001; *I*^2^ = 0.0%) and multivariate (*HR*: 8.94; 95% *CI*: 3.10–25.80; *P* < 0.001; *I*^2^ = 0.0%) analyses for DFS, the higher the pathological grade was, the worse the prognosis. In univariate (*HR*: 2.77; 95% *CI*: 0.91–8.41; *P* = 0.072; *I*^2^ = 53.8%) and multivariate (*HR*: 3.44; 95% *CI*: 0.80–14.75; *P* = 0.096; *I*^2^ = 62.1%) analyses for OS, the final result was not statistically significant.

#### EGFR and ALK mutations

Data were obtained from 3 [21, 24, 29] to 2 [20, 21] articles, respectively. There were no significant differences in OS (*HR*: 0.70; 95% *CI*: 0.35–1.41; *P* = 0.322; *I*^2^ = 0.0%) or DFS (*HR*: 0.52; 95% *CI*: 0.09–2.91; *P* = 0.459; *I*^2^ = 72.5%) between the EGFR mutation-positive and -negative groups in univariate analysis. In univariate (*HR*: 0.62; 95% *CI*: 0.13–3.03; *P* = 0.553; *I*^2^ = 60.0%) and multivariate (*HR*: 0.44; 95% *CI*: 0.04–4.49; *P* = 0.492; *I*^2^ = 76.4%) analyses for the ALK mutations, the results were not statistically significant between positive and negative patients for OS.

#### CT morphology

Three studies provided relevant data [27–29]. In univariate analysis, the OS of the patients with consolidative CT morphology was shorter than that with nodular CT morphology (*HR*: 3.11; 95% *CI*: 1.90–5.11; *P* < 0.001; *I*^2^ = 0.0%). The consolidation results for OS (*HR*: 3.44; 95% *CI*: 0.78–15.17; *P* = 0.102; *I*^2^ = 62.9%) and DFS (*HR*: 1.32; 95% *CI*: 0.15–11.74; *P* = 0.804; *I*^2^ = 79.3%) were not statistically significant in the multivariate analysis.

#### Pneumonia type

Three articles provided relevant data [22, 23, 25]. There was a worse prognosis for the patients with pneumonia type than solitary type in univariate (*HR*: 5.81; 95% *CI*: 3.68–9.19; *P* < 0.001; *I*^2^ = 0.0%) and multivariate (*HR*: 3.39; 95% *CI*: 1.19–9.62; *P* = 0.022; *I*^2^ = 3.4%) analyses for OS. Meanwhile, the trend of DFS was similar to that of OS in univariate (*HR*: 5.17; 95% *CI*: 3.31–8.08; *P* < 0.001; *I*^2^ = 37.4%) and multivariate (*HR*: 2.90; 95% *CI*: 1.55–5.42; *P* = 0.001; *I*^2^ = 19.3%) analyses.

#### Well-defined heterogeneous GGO

All data were from two studies [28, 29]. For OS, there was a shorter result for the patients with well-defined heterogeneous GGOs in univariate analysis (*HR*: 2.31; 95% *CI*: 1.27–4.19; *P* = 0.006; *I*^2^ = 0.0%), while there was no significant difference between the two groups in multivariate analysis (*HR*: 1.20; 95% *CI*: 0.44–3.25; *P* = 0.722; *I*^2^ = 0.0%).

#### Spiculated margin

Two studies provided relevant data [28, 29]. In univariate analysis for OS, patients with spiculated margins had a better prognosis (*HR*: 0.70; 95% *CI*: 0.54–0.92; *P* = 0.012; *I*^2^ = 0.0%).

#### Lobulated margin

Two studies were included [28, 29]. The results of the lobulated margin were not significantly different in the univariate analysis for OS (*HR*: 1.09; 95% *CI*: 0.66–1.80; *P* = 0.722; *I*^2^ = 0.0%).

#### Air bronchogram

Three studies provided relevant data [25, 28, 29]. In the univariate analysis for OS (*HR*: 1.40; 95% *CI*: 0.71–2.78; *P* = 0.336; *I*^2^ = 64.8%) and DFS (*HR*: 1.77; 95% *CI*: 0.55–5.67; *P* = 0.340; *I*^2^ = 85.1%), the pooled results for lobulated margins were not statistically significant.

## Discussion

This is the first systematic review and meta-analysis of all factors that potentially affect the prognosis of IMA. A total of 1062 patients from 11 studies were included, and 17 possible prognostic factors were identified. In univariate analysis, we found that gender, TNM stage, smoking history, lymph node metastasis, pleural metastasis, STAS, tumor size, CT findings of consolidative-type morphology, pneumonia type, and well-defined heterogeneous GGO were risk factors for death of IMA, while gender, age, smoking, lymph node metastasis, pathological grade, and pneumonia type sign were risk factors for recurrence, and spiculated margin sign was a protective factor for IMA. In multivariate analysis, smoking history, lymph node metastasis, STAS, tumor size, and pneumonia type sign were found to be risk factors for death in IMA, and lymph node metastasis, pathological grade, tumor size, and pneumonia type sign were risk factors for recurrence. The evidence for EGFR mutations, ALK mutations, lobulated margin sign, and air bronchogram sign as prognostic factors for IMA was insufficient.

### Summary of identified risk factors for IMA

Gender is an independent prognostic factor for lung adenocarcinoma. One study showed that women with lung adenocarcinoma generally had a better prognosis, which might be associated with estrogen receptor β overexpression in men [30–32]. However, as a special subtype, the prognostic guidance of gender for IMA is not yet fully understood. In this analysis, our results suggested that gender was a prognostic risk factor for IMA in univariate analysis (males had shorter OS and DFS than females).

Due to the inconsistency in the median age and tumor size provided by various studies, no group analysis was conducted. In most previous studies, the prognostic role of age and tumor size for IMA was highly controversial. In this meta-analysis, the results of the univariate analysis suggested that the risk of recurrence in elderly patients was higher. For tumor size, both in univariate and multivariate analyses, as well as OS and DFS, all results suggested that patients with larger tumors had poorer prognoses. However, the results of age and tumor size had significant heterogeneity, possibly due to less than 10 studies included, and the results of *I*^2^ may not be accurate [17]. Because of varying median values for age and tumor size, we can only consider age and tumor size as important prognostic risk factors for IMA, but further research is needed to determine the cutoff values. TNM stage is an important criterion to determine the stage of lung cancer. Only two studies were included in this analysis, and the pooled results suggested that stages III–IV had a higher risk of death than stages I–II in the univariate analysis. TNM stage can be used as an indicator to assess the risk of death in IMA, but the reliability needs to be further determined because of the small number of included studies. Most studies suggested that patients with lymph node and pleural metastasis had a worse prognosis, which was similar to our results [20, 25, 28, 29]. Lymph node metastasis further reflects the TNM stage, which can be combined to evaluate the prognosis of IMA.

Studies have shown that smoking is a major risk factor for lung cancer and can increase the incidence of lung cancer and mortality [33, 34]. In both univariate and multivariate analyses, the results showed that the risk of recurrence and death was higher in smokers. It is necessary for patients with IMA to quit smoking. Related health education and science popularization should also be further strengthened.

IMA is a type of cancer with special pathological manifestations that can also provide guidance for evaluating the prognosis of patients. According to histopathology, lung adenocarcinoma can be divided into three grades based on the degree of cell differentiation: well, moderately, and poorly differentiated. In this meta-analysis, the results suggested that patients with higher differentiation had a greater risk of recurrence. Therefore, for patients with advanced pathological grades, caution should be exercised, and appropriate adjustments should be made. In addition, IMA has a specific potential prognostic marker. STAS was recently recognized as a pattern of cancer invasion and a potential biomarker of poor prognosis in IMA [26, 35–38]. STAS is defined as micropapillary clusters, solid nests, or single cells spreading within air spaces beyond the edge of the main tumor [37]. In this analysis, the results showed that in both univariate and multivariate analyses, the OS of patients with STAS positivity was indeed shorter, and STAS can be considered a risk factor for mortality in IMA.

According to current studies, IMA has significant CT findings [39, 40]. Several studies have shown that the pneumonic-type sign has a worse prognosis than the solid-type sign [22, 23, 25]. In this study, our results also showed that it had a poorer prognosis. Besides, there has been evidence suggesting that consolidative-type morphology also is a risk prognostic indicator [26, 28, 29], our analysis indicated that it had a worse prognosis than nodular-type morphology. In addition, for OS in univariate analysis, CT findings of well-defined heterologous GGO were a risk prognostic factor. CT is a mature method routinely used in clinical practice for initial diagnosis and staging, guiding treatment, and monitoring cancer prediction [41, 42]. Consequently, the imaging performance of CT is greatly important for IMA.

For these prognostic risk factors mentioned above, we should give sufficient attention, and it is undoubtedly necessary to adopt more active treatment strategies and more rigorous clinical monitoring for patients with these factors.

### Productive factor

For CT manifestations of spiculated margins, the association with survival has not been well established for lung cancer [43]. Our results suggested that it was a protective factor for IMA, which might be confounded by patient conditions or other CT findings. Therefore, the protective effect should not be interpreted as a causal effect alone.

### Potential prognostic factors

IMA has a unique gene expression profile. KRAS mutations have been identified as the most common oncogenic driver in IMA (63–90%), followed by NRG1 fusions (7–27%) [6, 44–46]. IMA has a higher rate of ALK rearrangement mutations (2.2%) and a lower rate of EGFR mutations (0–5%) than nonmucinous adenocarcinoma of the lung [47–49]. KRAS, NRG1, EGFR, and ALK mutations are potential prognostic factors for IMA [20, 21, 23, 24, 28, 50]. Nevertheless, there are insufficient data to assess the association of KRAS and NRG1 mutations with prognosis, and conducting a meta-analysis is impossible. In this meta-analysis, none of the results was statistically significant. Therefore, EGFR and ALK mutations may not be prognostic protective factors for IMA. The mutation rates of both are low in IMA, and the number of samples that can be included in the study is very small, so more samples are needed for further study.

In addition, the results showed that CT signs of lobulated margins and air bronchograms were also not statistically significant. In conclusion, they could serve as potential prognostic factors, and further relevant studies could be conducted in the future to clarify their prognostic significance.

### Other factors not included

In addition to these prognostic factors, many other factors were not included in this meta-analysis due to a lack of data or insufficient research literature. Wang T. et al. suggested that the CT findings of location, spiculation, and tumor texture were associated with the prognosis of IMA, so we should pay attention to them when they appear in IMA [25]. The pathological features of IMA suggest that there are a wide range of mucins in the cells, and the expression of mucins may be of guiding significance for prognosis. A study reported that CD8 + tumor-infiltrating lymphocyte infiltration was associated with a shorter OS and worse prognosis in IMA [21]. According to a study, patients with abnormalities in the phosphatidylinositol 3-kinase (PI3K) signaling pathway displayed improved DFS, so an abnormal PI3K signaling pathway might be a protective factor for IMA [24]. Meanwhile, a study showed that acinar-predominant patients had a poorer prognosis than lepidic-predominant patients. Therefore, patients with acinar-predominant disease should be closely followed after surgery [19]. Oki et al*.* reported that the proportion of goblet cells was a pathological prognostic factor for IMA [27]. In addition, a study suggested that the type of palliative chemotherapy was related to the prognosis of late-stage IMA. Patients treated with immunotherapy may have better outcomes than those treated with other chemotherapies in IMA. This was useful for evaluating the therapeutic efficacy of patients [51]. Because IMA is a rare subtype, the sample size included in all studies is limited, which has a certain influence on the evaluation of prognostic factors. Therefore, more studies are needed to expand the sample size for further exploration.

Besides, a study showed that Mucin 5AC (MUC5AC) was associated with poor prognosis of IMA [52]. Wei L. et al. have found a hypersecretion of MUC5AC in patients with ILD through the determination of MUC5AC concentration in bronchoalveolar lavage fluid, and MUC5AC may be involved in the airway inflammatory response in ILD [53]. And a study reported that MUC5AC was significantly associated with the severity of ILD, and it could be a potential biomarker to predict the progression of ILD [54]. In conclusion, although there is no study describing the prognostic relationship between IMA and ILD, we should be more vigilant for patients with both ILD and IMA based on present research.

In addition to the abovementioned studies, we also focused on the similarities and differences in prognostic factors between the patients with IMA and non-IMA. There was a study proposing that micropapillary pattern was more common in IMA, and its prognosis was better than that of mixed IMA and non-IMA [55]. Besides, Gow C. H. et al. also showed that for stages I–II disease, the OS rate of patients with IMA was longer than that of patients with non-IMA [56]. But there was another study proposing that in patients with intrapulmonary recurrence, the prognosis of IMA was significantly worse than that of non-IMA [22]. Therefore, these studies indicated that there are significant differences in the prognosis of patients in IMA and non-IMA among different prognostic factors. However, there are still insufficient studies on the prognostic factors of IMA, and only a few studies have compared the prognostic factors between the patients with IMA and non-IMA. Thus, we did not collect enough data to compare the similarities and differences of prognostic factors further systematically between the patients with IMA and non-IMA, but we will continue to focus on this aspect in the future.

### Limitations

There are several limitations that need to be noted in this study. First, we found that some results exhibited significant heterogeneity, but due to the small number of articles included, we did not further analyze their sources. In the same way, potential publication bias was not further calculated. Second, the studies we included were all retrospective, with selection, missing data, or lack of endpoints, which may affect the assessment of prognostic survival factors. In addition, some important prognostic factors associated with IMA could not be analyzed because of insufficient data or an insufficient number of studies. We hope that more studies can fill these gaps. Finally, the accuracy of the results was affected to some extent because of the small number of articles included in each prognostic factor. Prognostic factors also influence each other, thereby affecting the final outcome.

## Conclusions

In this systematic review and meta-analysis, we comprehensively evaluated prognostic factors for IMA. In univariate and multivariate analyses, we identified 12 risk factors and 1 protective factor for IMA. We believe that the presented findings would be considerably helpful in daily clinical practice.

### Supplementary Information

**Additional file 1.** The PRISMA checklist.

**Additional file 2.** The detailed search strategy of each database.

**Additional file 3.** The forest plots of each factor.

## Data Availability

All the data that support the conclusions of this study can be obtained from the corresponding author.

## Abbreviations

IMA: Invasive mucinous adenocarcinoma of the lung
HR: Hazard ratio
CI: Confidence intervals
OS: Overall survival
DFS: Disease-free survival
CT: Computed tomography
STAS: Spread through air spaces
GGO: Ground-glass opacity
EGFR: Epidermal growth factor receptor
ALK: Anaplastic lymphoma kinase
ERS: European Respiratory Society
PRISMA: Preferred Reporting Items for Systematic Reviews and Meta-Analyses
ATS: American Thoracic Society
IASLC: International Association for Cancer Research
NOS: Newcastle–Ottawa Quality Assessment Scale
PI3K: Phosphatidylinositol 3-kinase
MUC5AC: Mucin 5AC
ILD: Interstitial lung disease
