# Supplementary material for: Prognostic factors for invasive mucinous adenocarcinoma of the lung: systematic review and meta-analysis
Source: World J Surg Oncol. 2024 Feb 2;22:41. doi: 10.1186/s12957-024-03326-4 (PMC10835932; doi:10.1186/s12957-024-03326-4)
Supplement: Supplementary file 1 — Additional file 1. The PRISMA checklist. [file 12957_2024_3326_MOESM1_ESM.docx]

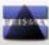
 **PRISMA 2020 Checklist**

| **Section/topic** | **Item**  **No** | **Checklist item** | **Reported on Page Number/Line**  **Number** | **Reported on**  **Section/Paragraph** |
| --- | --- | --- | --- | --- |
| **TITLE** | | | | |
| Title | 1 | Identify the report as a systematic review. | Page 1/Lines 1-2 | Title |
| **ABSTRACT** | | | | |
| Abstract | 2 | See the PRISMA 2020 for Abstracts checklist (Table 2). | Pages 1-2/Lines 17-37 | Abstract |
| **INTRODUCTION** | | | | |
| Rationale | 3 | Describe the rationale for the review in the context of existing knowledge. | Page 2-3/Lines 41-50 | Introduction |
| Objectives | 4 | Provide an explicit statement of the objective(s) or question(s) the review addresses. | Page 3/Lines 51-64 | Introduction |
| **METHODS** | | | | |
| Eligibility criteria | 5 | Specify the inclusion and exclusion criteria for the review and how studies were grouped for the syntheses. | Page 4/Lines 76-85 | Materials and methods/Paragraph 2 |
| Information  sources | 6 | Specify all databases, registers, websites, organisations, reference lists and other sources searched or consulted to identify studies. Specify the date when each source was last searched or consulted. | Page 4/Lines 71-74 | Materials and methods/Paragraph 1 |
| Search strategy | 7 | Present the full search strategies for all databases, registers and websites, including any filters and limits used. | Page 4/Lines 71-74 | Materials and methods/Paragraph 1 |
| Selection process | 8 | Specify the methods used to decide whether a study met the inclusion criteria of the review, including how many reviewers screened each record and each report retrieved, whether they worked independently, and if applicable, details of automation tools used in the process. | Page 4-5/Lines 87-90 | Materials and methods/Paragraph 3 |
| Data collection  process | 9 | Specify the methods used to collect data from reports, including how many reviewers collected data from each  report, whether they worked independently, any processes for obtaining or confirming data from study investigators, and if applicable, details of automation tools used in the process. | Page 4/Lines 87-90 | Materials and methods/Paragraph 3 |
| Data items | 10a | List and define all outcomes for which data were sought. Specify whether all results that were compatible with each outcome domain in each study were sought (e.g. for all measures, time points, analyses), and if not, the methods  used to decide which results to collect. | Page 4/Lines 89-90 | Materials and methods/Paragraph 3 |
|  | 10b | List and define all other variables for which data were sought (e.g. participant and intervention characteristics, funding sources). Describe any assumptions made about any missing or unclear information. | N/A | N/A |

| Study risk of bias assessment | 11 | Specify the methods used to assess risk of bias in the included studies, including details of the tool(s) used,  how many reviewers assessed each study and whether they worked independently, and if applicable, details of automation tools used in the process. | Page 5/Lines 107-108 | Materials and methods/Paragraph 4 |
| --- | --- | --- | --- | --- |
| Effect measures | 12 | Specify for each outcome the effect measure(s) (e.g. risk ratio, mean difference) used in the synthesis or presentation of results. | Page 5/Lines 97-100 | Materials and methods/Paragraph 4 |
| Synthesis methods | 13a | Describe the processes used to decide which studies were eligible for each synthesis. | Page 6/Lines 111-115 | Results/Paragraph 1 |
|  | 13b | Describe any methods required to prepare the data for presentation or synthesis, such as handling of missing summary statistics, or data conversions. | N/A | N/A |
|  | 13c | Describe any methods used to tabulate or visually display results of individual studies and syntheses. | Page 5/Lines 97-100 | Materials and methods/Paragraph 4 |
|  | 13d | Describe any methods used to synthesize results and provide a rationale for the choice(s). If meta-analysis was performed, describe the model(s), method(s) to identify the presence and extent of statistical heterogeneity, and software package(s) used. | Page 5/Lines 100-106 | Materials and methods/Paragraph 4 |
|  | 13e | Describe any methods used to explore possible causes of heterogeneity among study results. | Page 5/Lines 103-106 | Materials and methods/Paragraph 4 |
|  | 13f | Describe any sensitivity analyses conducted to assess robustness of the synthesized results. | Page 5/Lines 103-105 | Materials and methods/Paragraph 4 |
| Reporting bias  assessment | 14 | Describe any methods used to assess risk of bias due to missing results in a synthesis (arising from reporting biases). | Page 5/Lines 107-108 | Materials and methods/Paragraph 4 |
| Certainty  assessment | 15 | Describe any methods used to assess certainty (or confidence) in the body of evidence for an outcome. | N/A | N/A |
| **RESULTS** | | | | |
| Study selection | 16a | Describe the results of the search and selection process, from the number of records identified in the search to the number of studies included in the review, ideally using a flow diagram. | Page 6/Lines 111-115 | Results/Paragraph 1 |
|  | 16b | Cite studies that met many but not all inclusion criteria (‘near-misses’) and explain why they were excluded. | Page 6/Lines 111-113 | Results/Paragraph 1 |
| Study  characteristics | 17 | Cite each included study and present its characteristics. | Page 6-7/Lines 118-121 | Results/Paragraph 2 |
| Risk of bias in  studies | 18 | Present assessments of risk of bias for each included study. | N/A | N/A |
| Results of  individual studies | 19 | For all outcomes, present, for each study: (a) summary statistics for each group (where appropriate) and (b) an effect estimate and its precision (e.g. confidence/credible interval), ideally using structured tables or plots. | Page 7-11/Lines 126-201 | Results/Paragraph 3 |

5-2

| Results of  syntheses | 20a | For each synthesis, briefly summarise the characteristics and risk of bias among contributing studies. | N/A | N/A |
| --- | --- | --- | --- | --- |
|  | 20b | Present results of all statistical syntheses conducted. If meta-analysis was done, present for each the summary  estimate and its precision (e.g. confidence/credible interval) and measures of statistical heterogeneity. If comparing groups, describe the direction of the effect. | Page 8-11/Lines 129-201 | Results/Paragraph 3 |
|  | 20c | Present results of all investigations of possible causes of heterogeneity among study results. | Page 8-11/Lines 129-201 | Results/Paragraph 3 |
|  | 20d | Present results of all sensitivity analyses conducted to assess the robustness of the synthesized results. | N/A | N/A |
| Reporting biases | 21 | Present assessments of risk of bias due to missing results (arising from reporting biases) for each synthesis assessed. | N/A | N/A |
| Certainty of  evidence | 22 | Present assessments of certainty (or confidence) in the body of evidence for each outcome assessed. | N/A | N/A |
| **DISCUSSION** | | | | |
| Discussion | 23a | Provide a general interpretation of the results in the context of other evidence. | Page 11-15/Lines 203-283 | Discussion/Paragraph 1-5 |
|  | 23b | Discuss any limitations of the evidence included in the review. | Page 17/Lines 325-334 | Discussion/Paragraph 6 |
|  | 23c | Discuss any limitations of the review processes used. | Page 17/Lines 325-334 | Discussion/Paragraph 6 |
|  | 23d | Discuss implications of the results for practice, policy, and future research. | Page 17/Lines 336-339 | Discussion/Paragraph 7 |
| **OTHER INFORMATION** | | | | |
| Registration and protocol | 24a | Provide registration information for the review, including register name and registration number, or state that the review was not registered. | There was no registration for this study | There was no registration for this study |
|  | 24b | Indicate where the review protocol can be accessed, or state that a protocol was not prepared. | There was no protocol for this study | There was no protocol for this study |
|  | 24c | Describe and explain any amendments to information provided at registration or in the protocol. | N/A | N/A |
| Support | 25 | Describe sources of financial or non-financial support for the review, and the role of the funders or sponsors in the review. | Page 19/Lines 370-372 | Discussion/Paragraph 13 |
| Competing  interests | 26 | Declare any competing interests of review authors. | Page 19/Lines 368 | Discussion/Paragraph 12 |
| Availability of data, code and other  materials | 27 | Report which of the following are publicly available and where they can be found: template data collection forms; data extracted from included studies; data used for all analyses; analytic code; any other materials used in the  review. | Not report in this article | Not report in this article |

5-3

**Table 2 PRISMA 2020 for Abstracts checklist**

| **Section/topic** | **Item**  **No** | **Checklist item** | **Reported on Page Number/Line**  **Number** | **Reported on**  **Section/Paragraph** |
| --- | --- | --- | --- | --- |
| **TITLE** | | | | |
| Title | 1 | Identify the report as a systematic review. | Page 1/Lines 1-2 | Title |
| **BACKGROUND** | | | | |
| Objectives | 2 | Provide an explicit statement of the main objective(s) or question(s) the review addresses. | Page 2-3/Lines 41-45 | Introduction |
| **METHODS** | | | | |
| Eligibility criteria | 3 | Specify the inclusion and exclusion criteria for the review. | Page 4/Lines 73-82 | Materials and methods/Paragraph 2 |
| Information  sources | 4 | Specify the information sources (e.g. databases, registers) used to identify studies and the date when each was last searched. | Page 4/Lines 71-74 | Materials and methods/Paragraph 1 |
| Risk of bias | 5 | Specify the methods used to assess risk of bias in the included studies. | Page 5/Lines 107-108 | Materials and methods/Paragraph 4 |
| Synthesis of  results | 6 | Specify the methods used to present and synthesize results. | Page 5/Lines 97-99 | Materials and methods/Paragraph 4 |
| **RESULTS** | | | | |
| Included studies | 7 | Give the total number of included studies and participants and summarise relevant characteristics of studies. | Page 6/Lines 111-115 | Results/Paragraph 1 |
| Synthesis of  results | 8 | Present results for main outcomes, preferably indicating the number of included studies and participants for each. If meta-analysis was done, report the summary estimate and confidence/credible interval. If comparing groups,  indicate the direction of the effect (i.e. which group is favoured). | Page 8-11/Lines 126-201 | Results/Paragraph 3 |
| **DISCUSSION** | | | | |
| Limitations of  evidence | 9 | Provide a brief summary of the limitations of the evidence included in the review (e.g. study risk of bias, inconsistency and imprecision). | Page 17/Lines 325-334 | Discussion/Paragraph 6 |
| Interpretation | 10 | Provide a general interpretation of the results and important implications. | Page 11-15/Lines 203-283 | Discussion/Paragraph 1-5 |
| **OTHER** | | | | |
| Funding | 11 | Specify the primary source of funding for the review. | Page 19/Lines 370-372 | Discussion/Paragraph 13 |
| Registration | 12 | Provide the register name and registration number. | There was no registration for this study | There was no registration for this study |

5-4

Updated on September 21, 2020
