# Supplementary material for: Prognostic factors for invasive mucinous adenocarcinoma of the lung: systematic review and meta-analysis
Source: World J Surg Oncol. 2024 Feb 2;22:41. doi: 10.1186/s12957-024-03326-4 (PMC10835932; doi:10.1186/s12957-024-03326-4)
Supplement: Supplementary file 2 — Additional file 2. The detailed search strategy of each database. [file 12957_2024_3326_MOESM2_ESM.docx]

**Additional file 2. The detailed search strategy of each database.**

1. **Search strategy for PubMed:**

(((invasive mucinous adenocarcinoma[Title/Abstract])) AND (lung[Title/Abstract])) AND (prognosis[Title/Abstract]) OR (((invasive mucinous adenocarcinoma[Title/Abstract])) AND (lung[Title/Abstract])) AND (prognostic[Title/Abstract])

1. **Search strategy for Embase:**

('Invasive mucinous adenocarcinoma'):ti,ab,kw AND ((lung):ti,ab,kw) AND ((prognosis):ti,ab,kw) OR ('Invasive mucinous adenocarcinoma'):ti,ab,kw AND ((lung):ti,ab,kw) AND ((prognostic):ti,ab,kw)

1. **Search strategy for Cochrane Library:**

(invasive mucinous adenocarcinoma and lung and prognosis) OR (invasive mucinous adenocarcinoma and lung and prognostic) in Title Abstract Keyword

1. **Search strategy for Web of Science:**

(invasive mucinous adenocarcinoma and lung and prognosis) OR (invasive mucinous adenocarcinoma and lung and prognostic) (Topic)
