# Supplementary material for: Prognostic factors for invasive mucinous adenocarcinoma of the lung: systematic review and meta-analysis
Source: World J Surg Oncol. 2024 Feb 2;22:41. doi: 10.1186/s12957-024-03326-4 (PMC10835932; doi:10.1186/s12957-024-03326-4)
Supplement: Supplementary file 3 — Additional file 3. The forest plots of each factor. [file 12957_2024_3326_MOESM3_ESM.docx]

**Supplementary figure 1-4:** The forest plots of each factor.

Supplementary figures 1A-Q: Forest plot of the meta-analysis in univariate analysis of OS

Supplementary figure 1A: Forest plot of gender


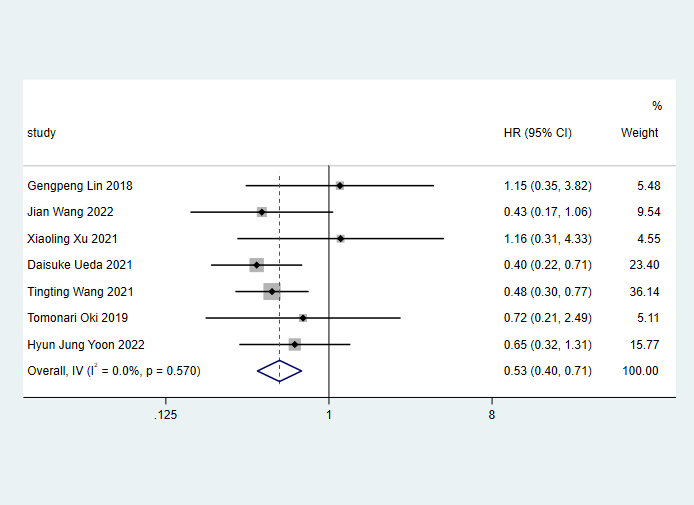


Supplementary figure 1B: Forest plot of age


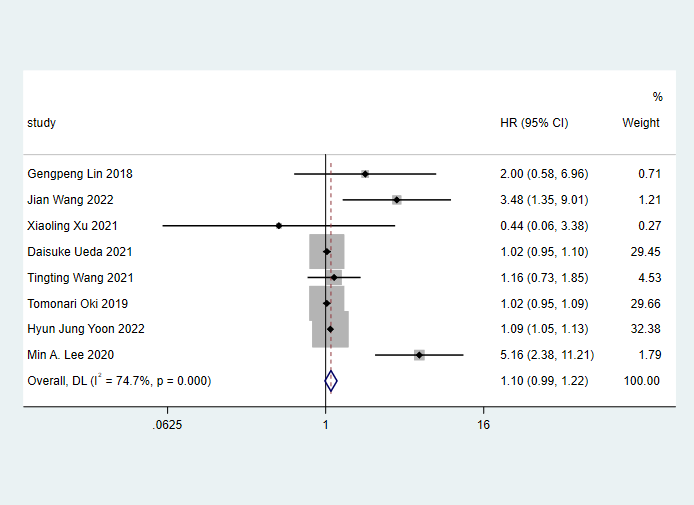


Supplementary figure 1C: Forest plot of TNM stage


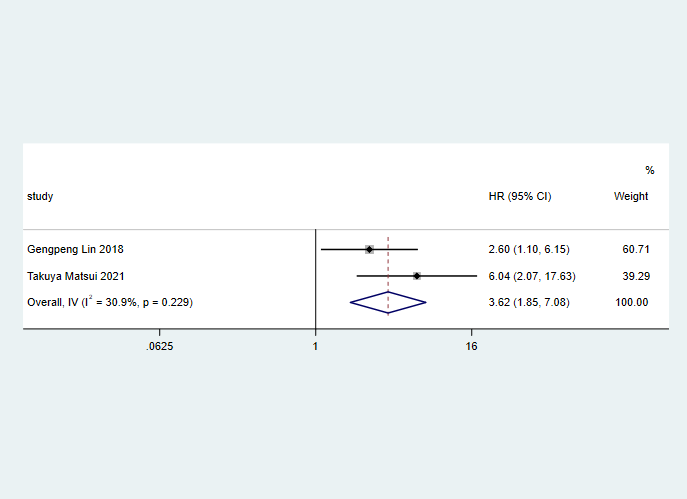


Supplementary figure 1D: Forest plot of smoking status


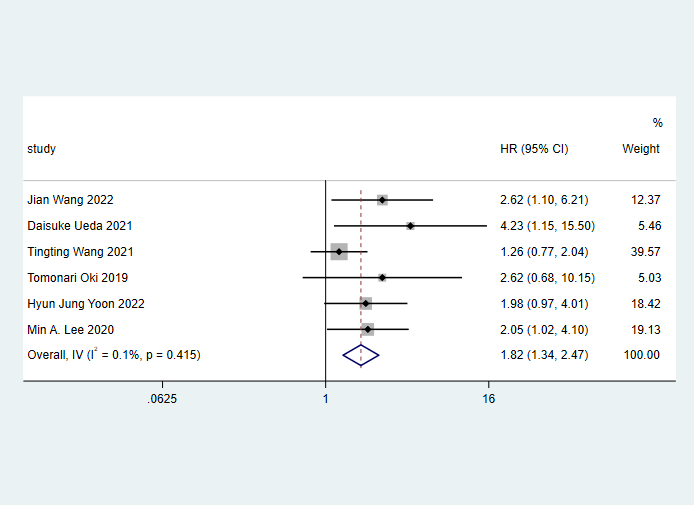


Supplementary figure 1E: Forest plot of lymph node metastasis


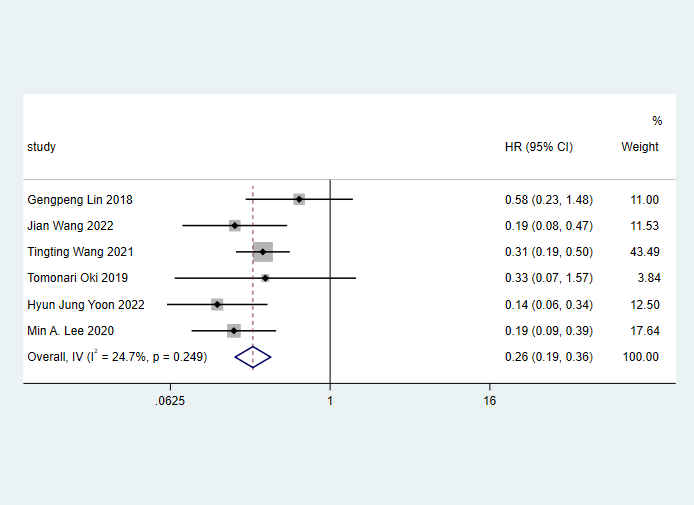


Supplementary figure 1F: Forest plot of pleural invasion


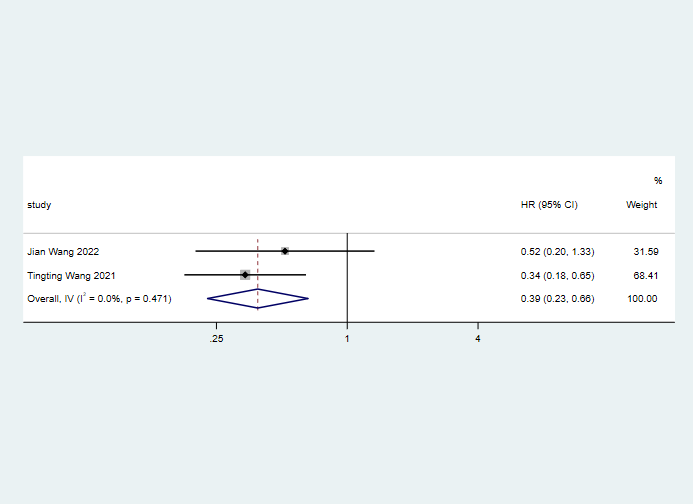


Supplementary figure 1G: Forest plot of STAS


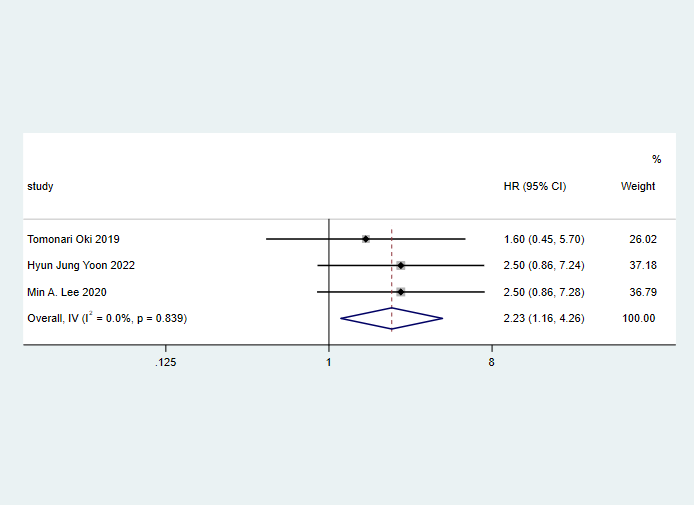


Supplementary figure 1H: Forest plot of pathological grade


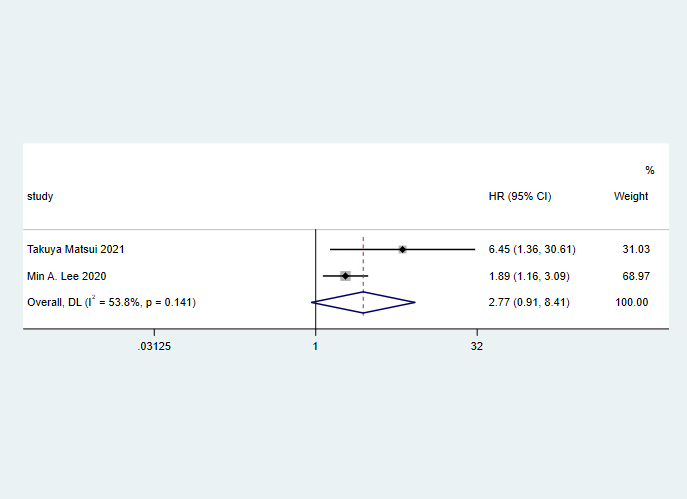


Supplementary figure 1I: Forest plot of EGFR mutations


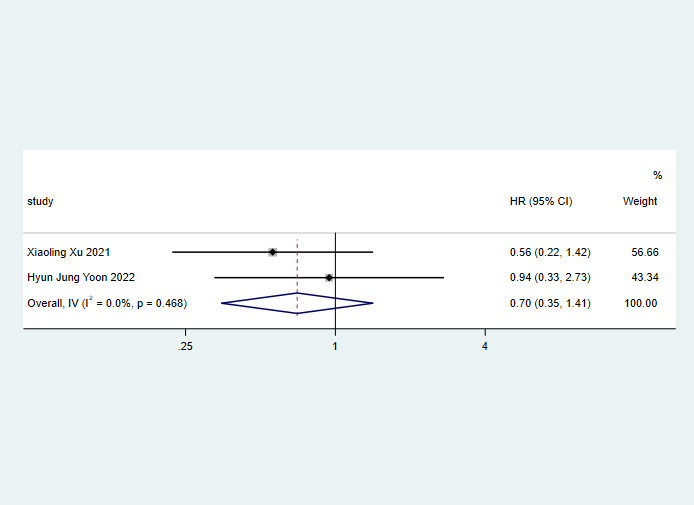


Supplementary figure 1J: Forest plot of ALK mutations


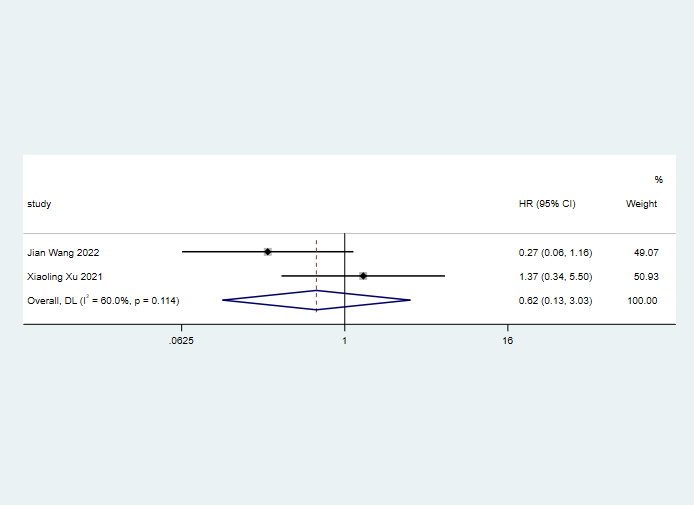


Supplementary figure 1K: Forest plot of tumor size


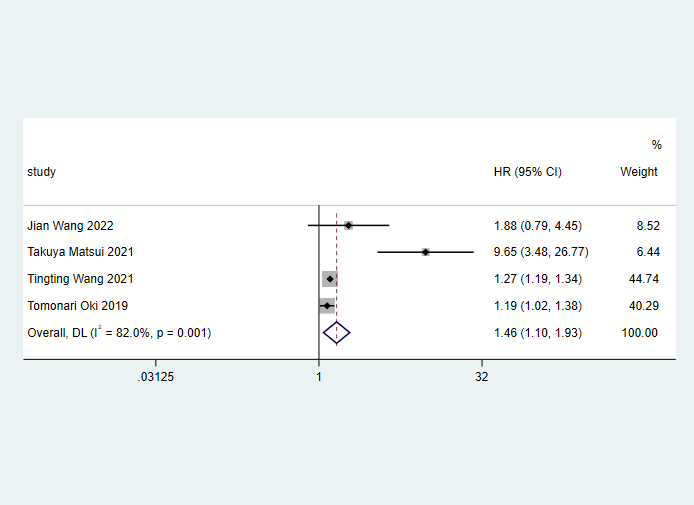


Supplementary figure 1L: Forest plot of CT morphology


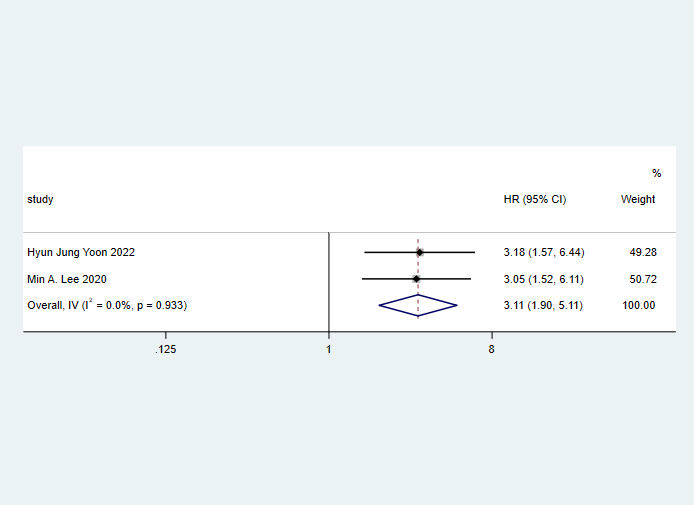


Supplementary figure 1M: Forest plot of pneumonia type


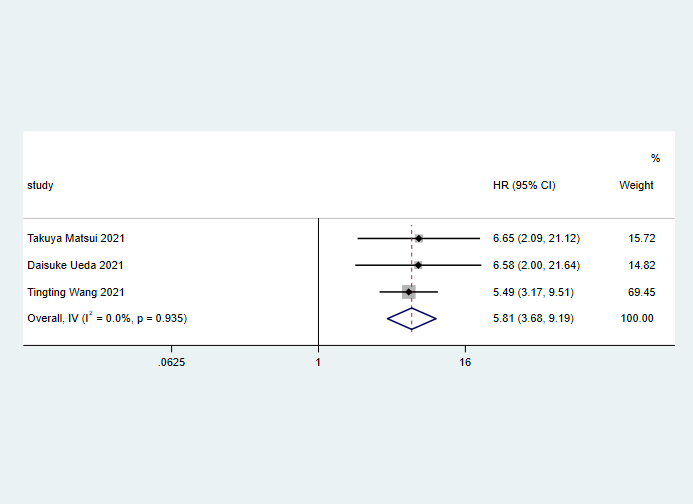


Supplementary figure 1N: Forest plot of well-defined heterogeneous GGOs


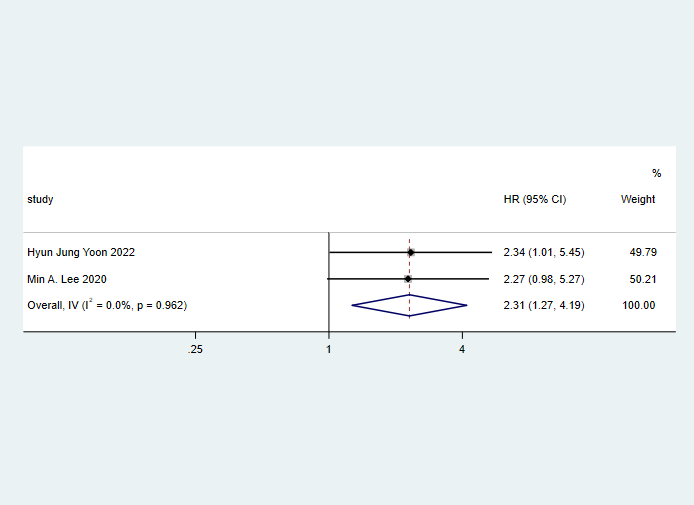


Supplementary figure 1O: Forest plot of spiculated margin


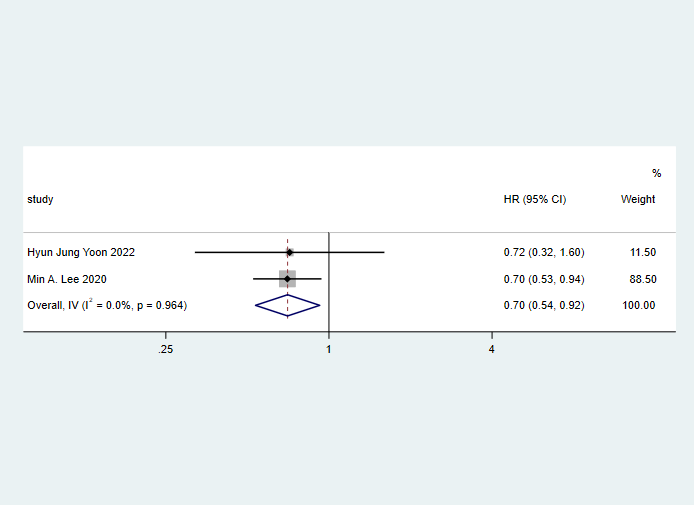


Supplementary figure 1P: Forest plot of lobulated margin


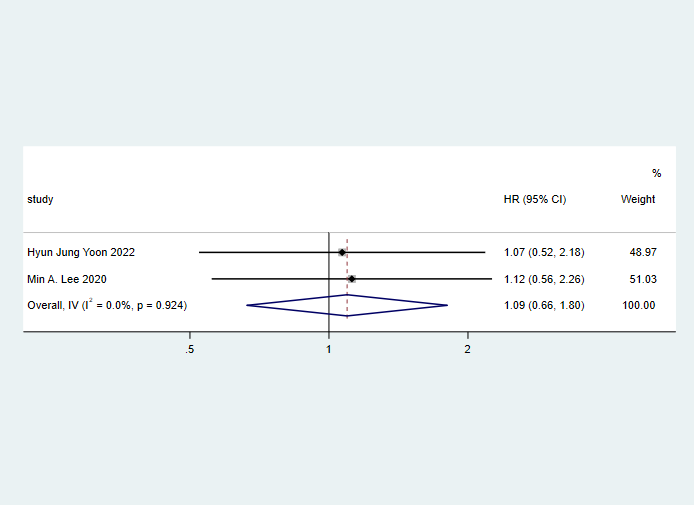


Supplementary figure 1Q: Forest plot of air bronchogram


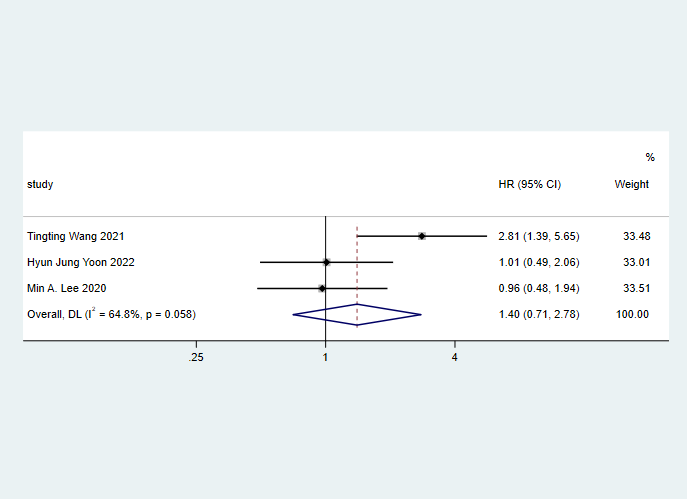


Supplementary figures 2A-K: Forest plot of the meta-analysis in multivariate analysis of OS

Supplementary figure 2A: Forest plot of gender


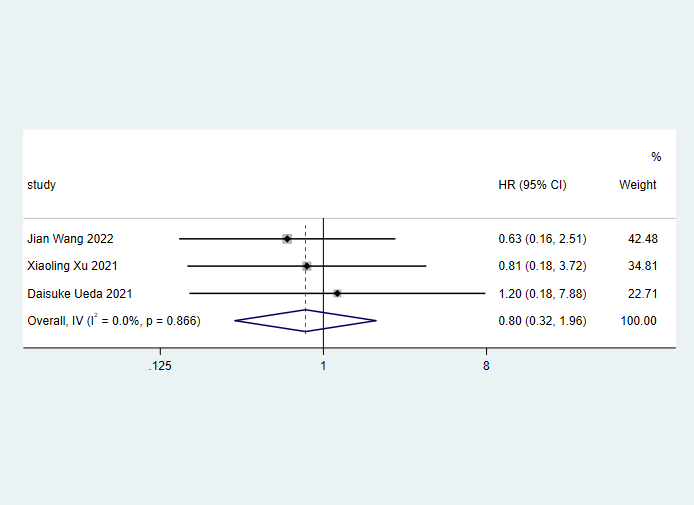


Supplementary figure 2B: Forest plot of age


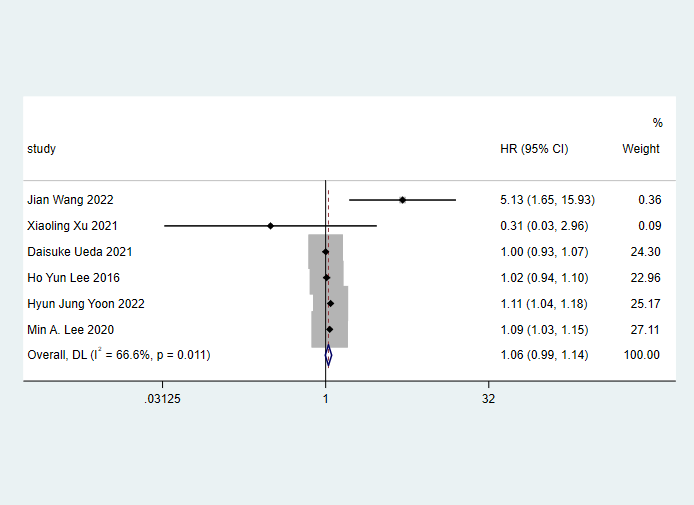


Supplementary figure 2C: Forest plot of smoking status


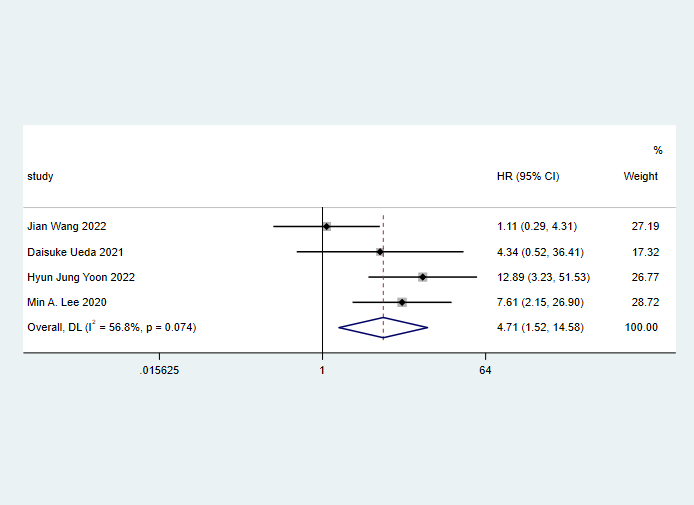


Supplementary figure 2D: Forest plot of lymph node metastasis


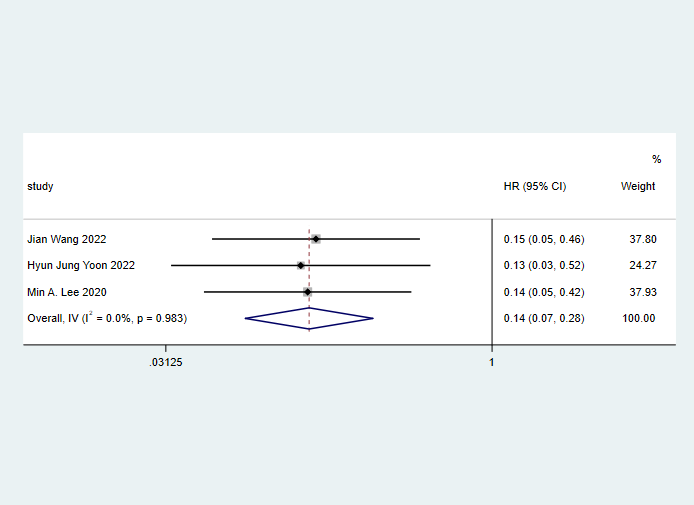


Supplementary figure 2E: Forest plot of STAS


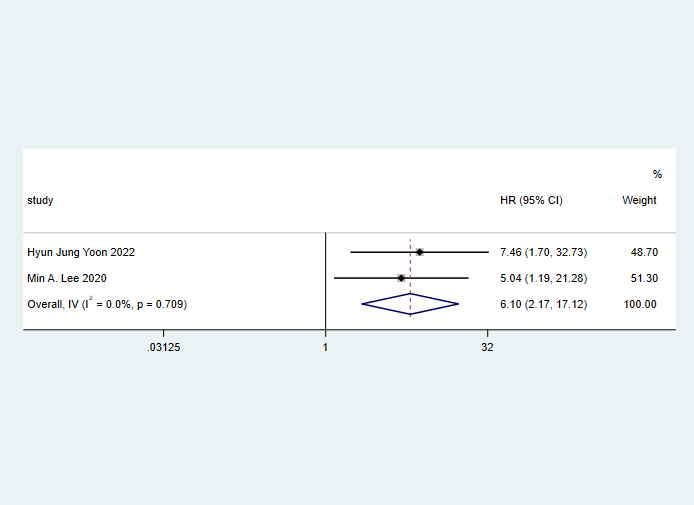


Supplementary figure 2F: Forest plot of pathological grade


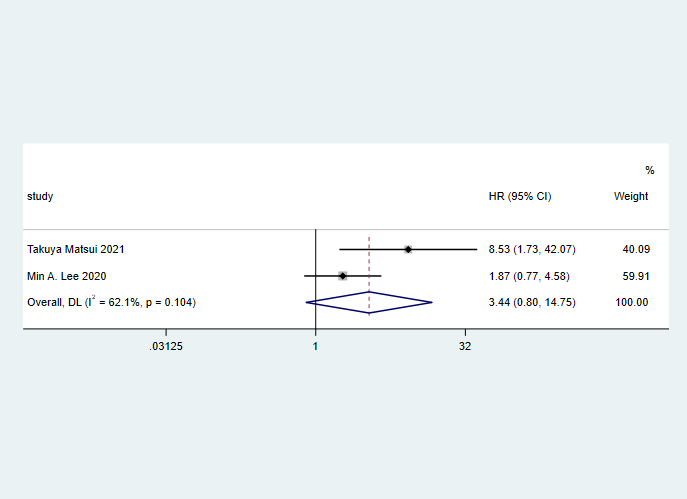


Supplementary figure 2G: Forest plot of ALK mutations


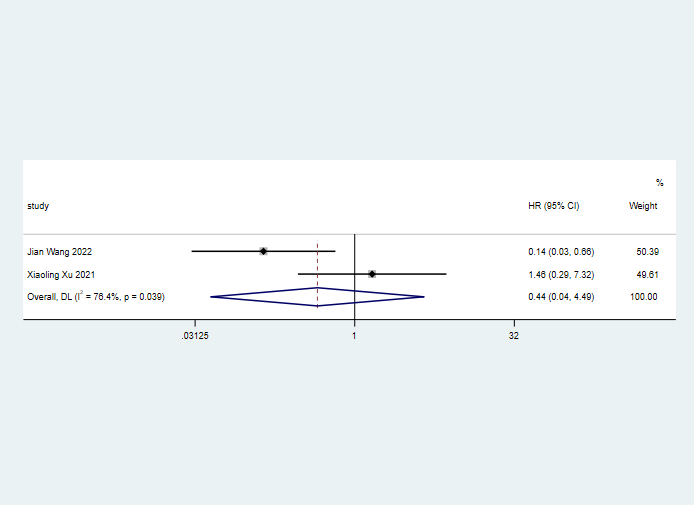


Supplementary figure 2H: Forest plot of tumor size


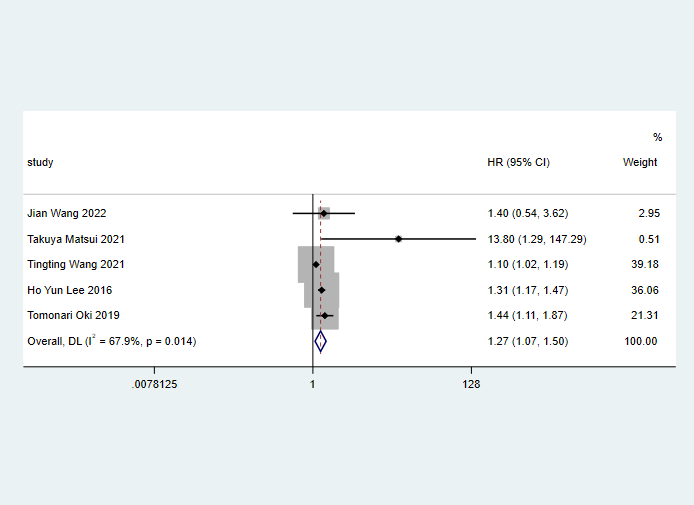


Supplementary figure 2I: Forest plot of CT morphology


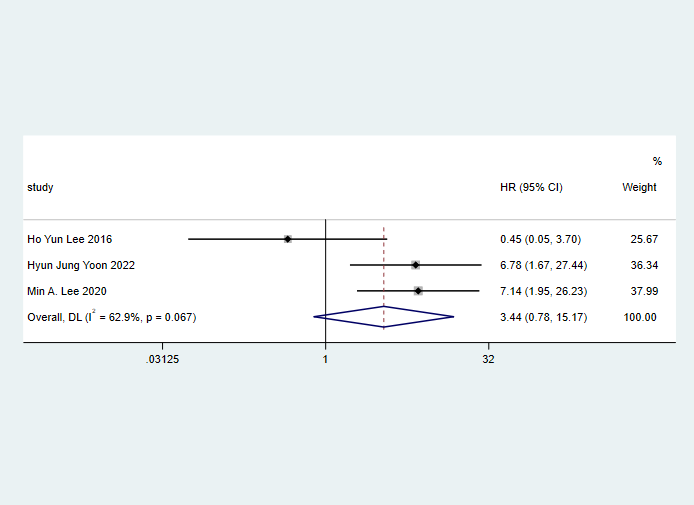


Supplementary figure 2J: Forest plot of pneumonia type


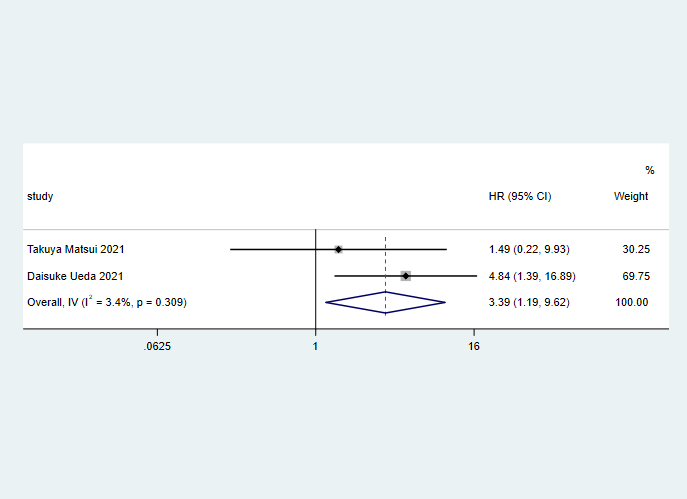


Supplementary figure 2K: Forest plot of well-defined heterogeneous GGOs


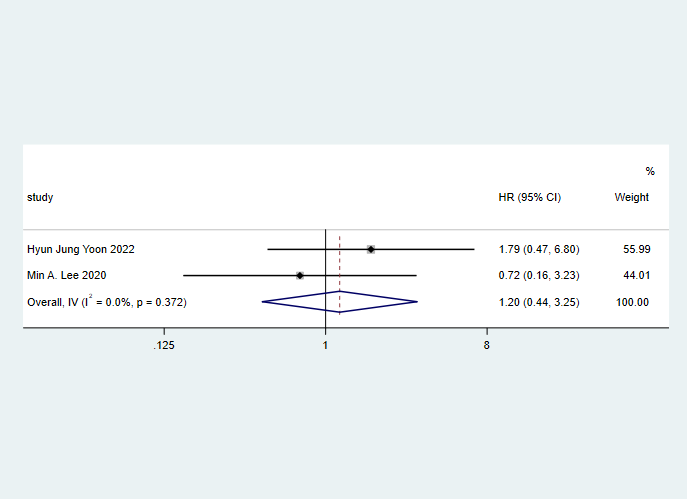


Supplementary figures 3A-J: Forest plot of the meta-analysis in univariate analysis of the DFS

Supplementary figure 3A: Forest plot of gender


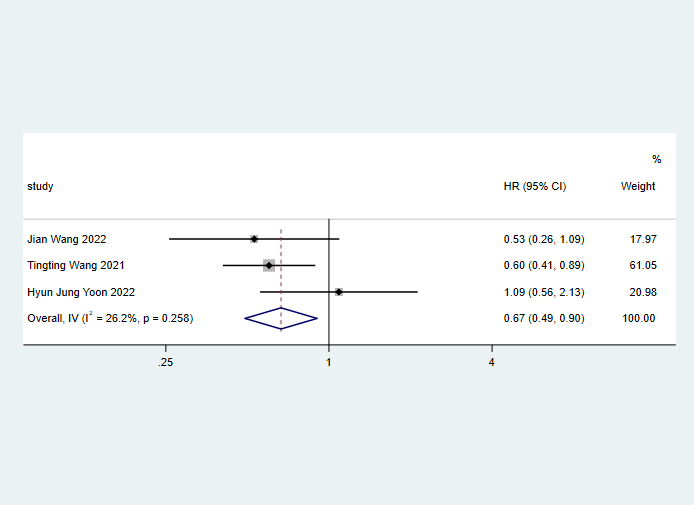


Supplementary figure 3B: Forest plot of age


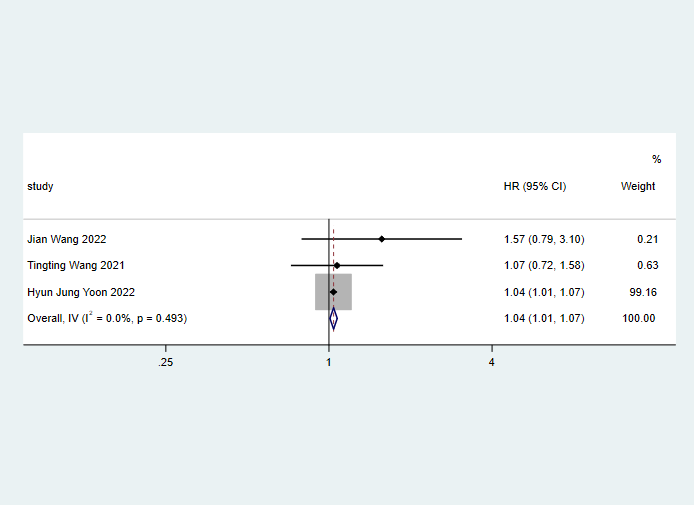


Supplementary figure 3C: Forest plot of smoking status


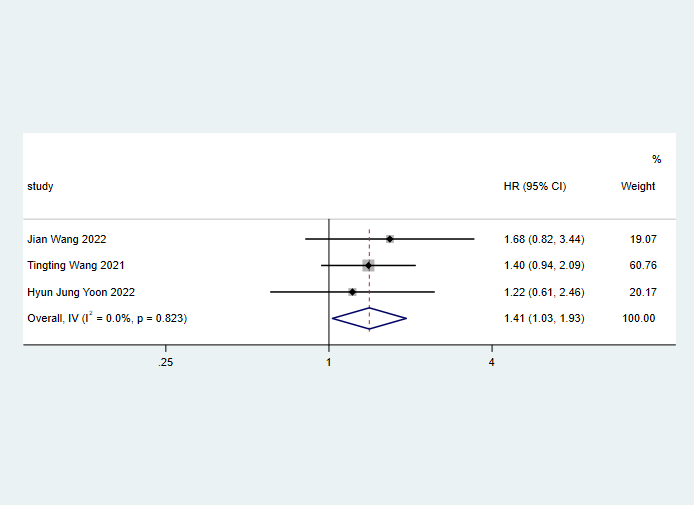


Supplementary figure 3D: Forest plot of lymph node metastasis


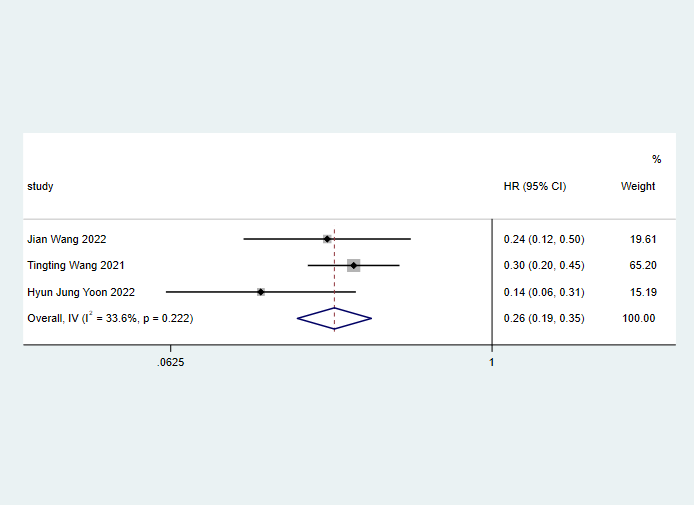


Supplementary figure 3E: Forest plot of pleural invasion


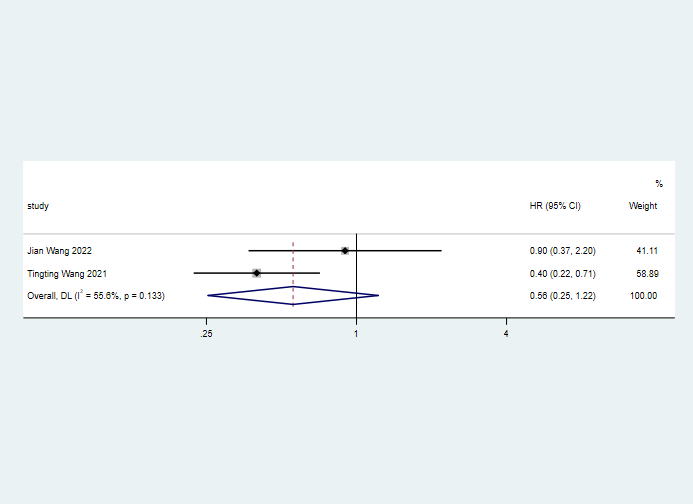


Supplementary figure 3F: Forest plot of pathological grade


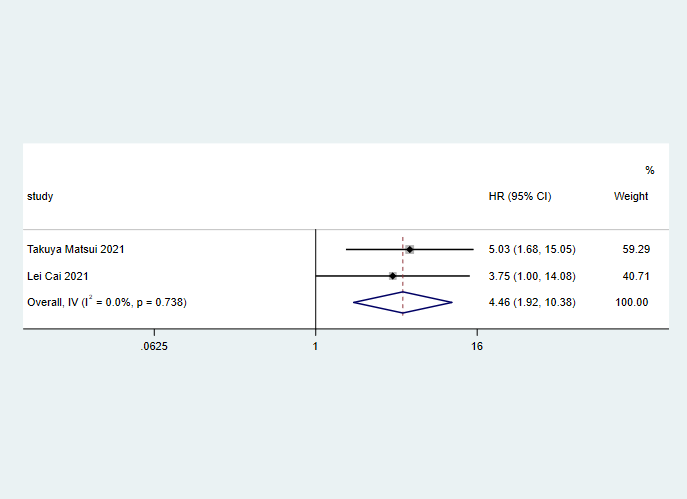


Supplementary figure 3G: Forest plot of EGFR mutations


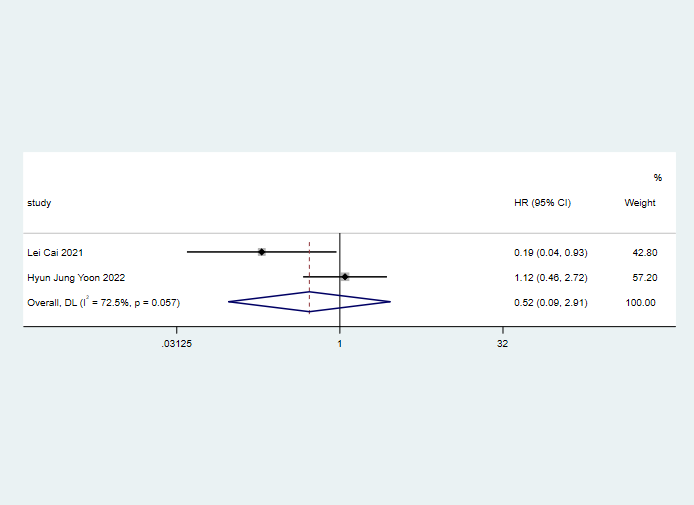


Supplementary figure 3H: Forest plot of tumor size

**
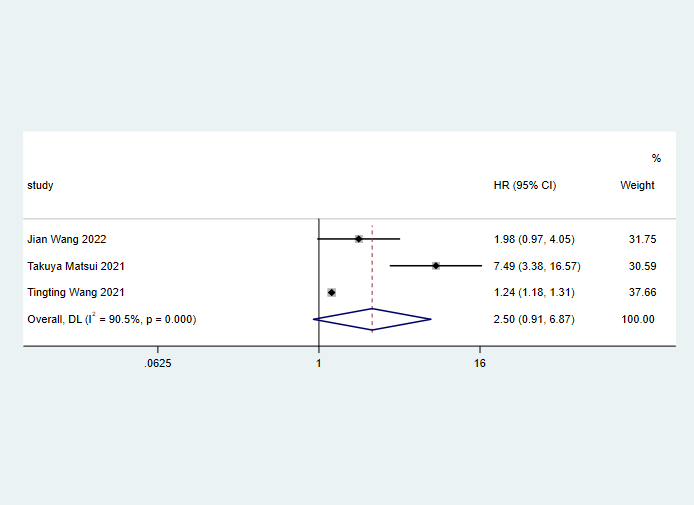
**

Supplementary figure 3I: Forest plot of pneumonia type

**
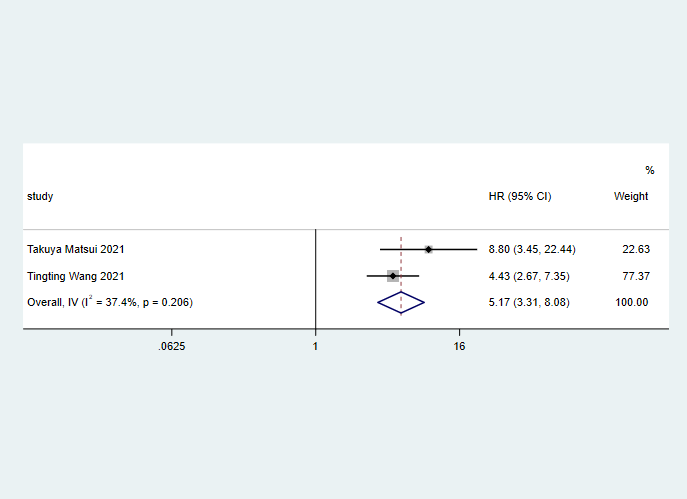
**

Supplementary figure 3J: Forest plot of air bronchogram

**
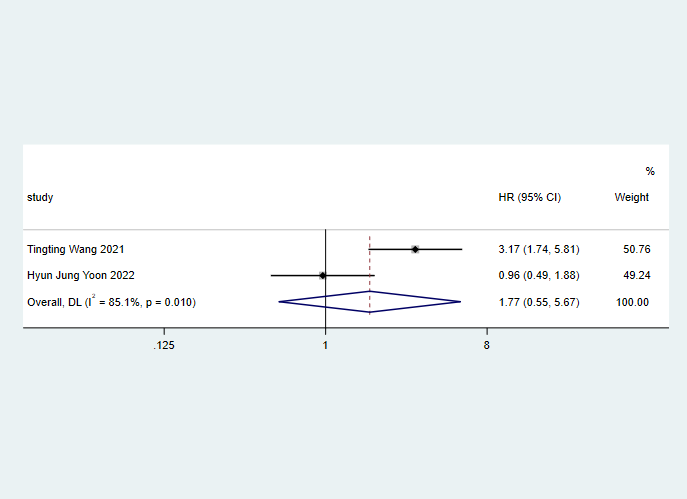
**

Supplementary figures 4A-G: Forest plot of the meta-analysis in multivariate analysis of DFS

Supplementary figure 4A: Forest plot of age

**
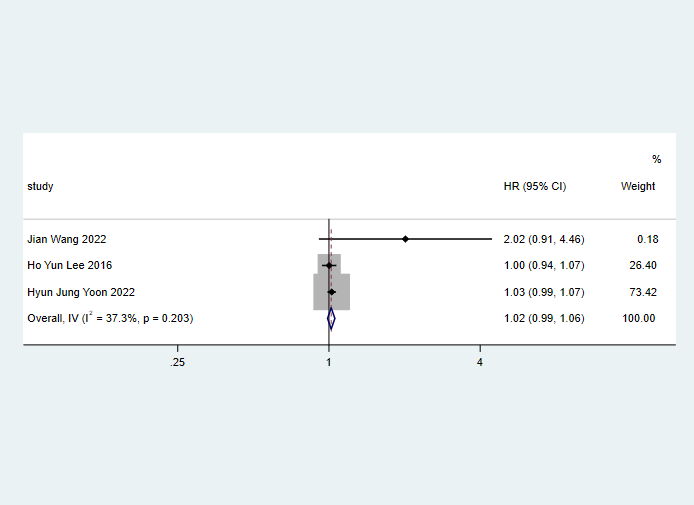
**

Supplementary figure 4B: Forest plot of smoking status

**
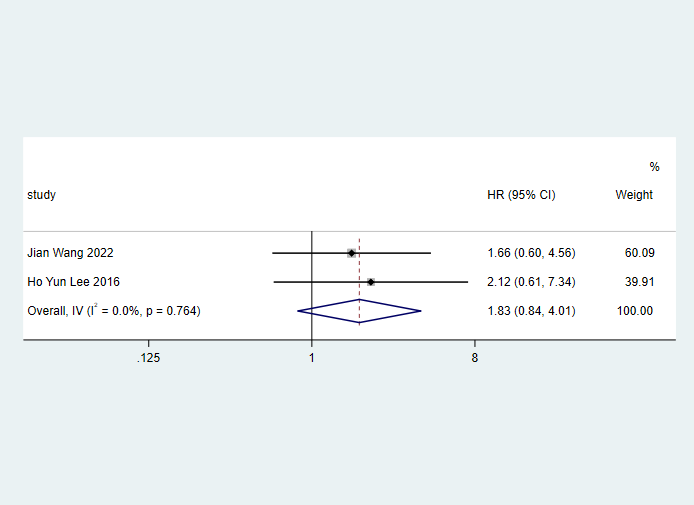
**

Supplementary figure 4C: Forest plot of lymph node metastasis

**
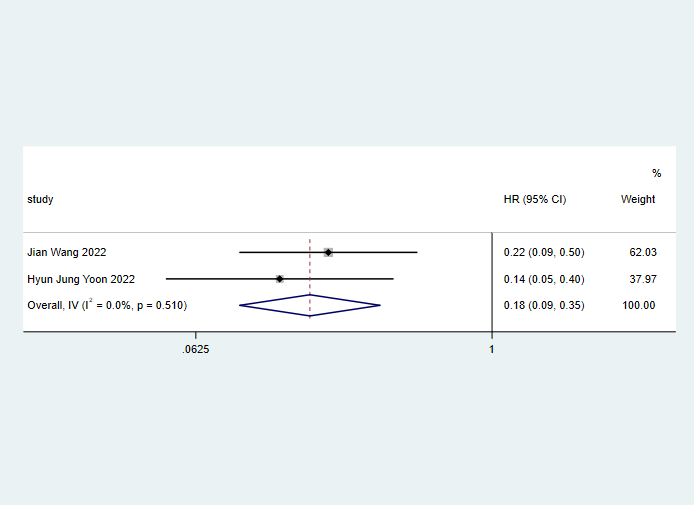
**

Supplementary figure 4D: Forest plot of pathological grade

**
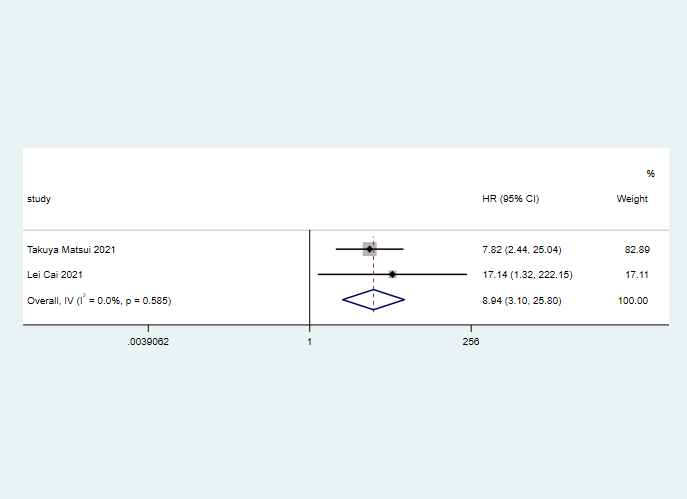
**

Supplementary figure 4E: Forest plot of tumor size

**
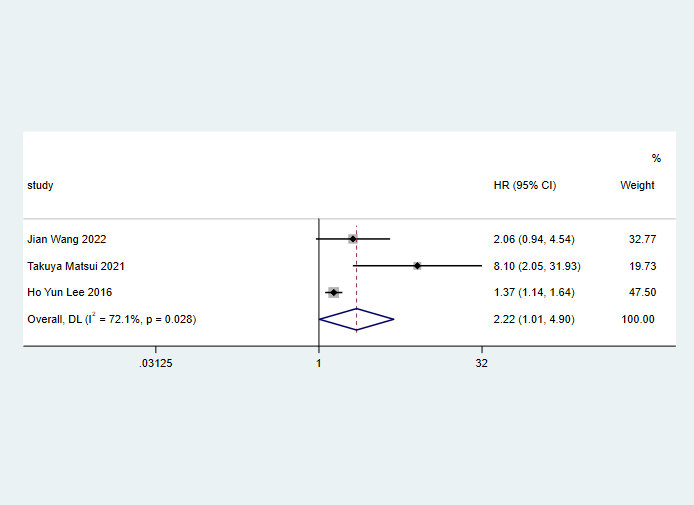
**

Supplementary figure 4F: Forest plot of CT morphology

**
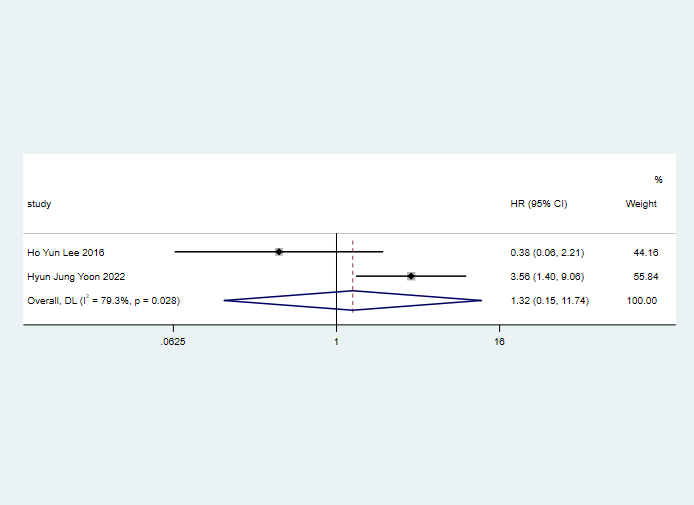
**

Supplementary figure 4G: Forest plot of pneumonia type

**
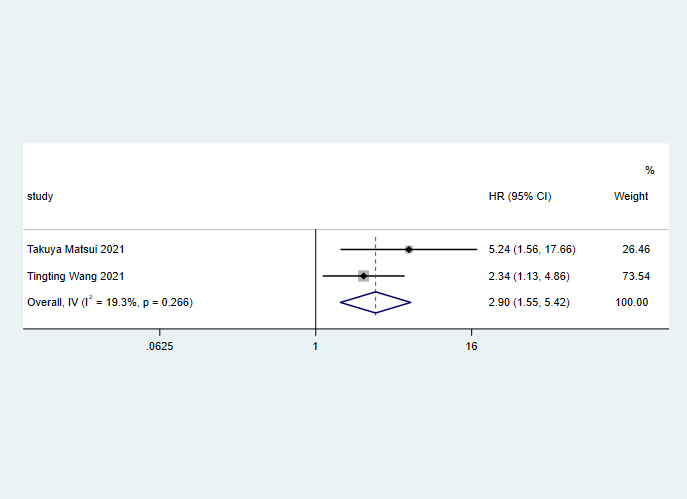
**
